# Flexor tendon avulsion and PIP joint fracture-dislocation in a 17-year-old football player: A case report

**DOI:** 10.1016/j.jpra.2025.12.015

**Published:** 2025-12-19

**Authors:** Summer Aldabbeh, Taylor Calicchia, Andrew Esterle

**Affiliations:** a Department of Surgery, Cleveland Clinic Akron General, Akron, OH; b Department of Orthopaedic Surgery, Cleveland Clinic Akron General, Akron, OH

**Keywords:** PIP joint fracture-dislocation, Reconstruction, Sports injury, Tendon avulsion, Tendon repair

## Abstract

We present a rare case of flexor digitorum superficialis (FDS) avulsion and dorsal fracture-dislocation of the proximal interphalangeal (PIP) joint with an intact flexor digitorum profundus (FDP) in a 17-year-old high school football player. This unusual injury pattern, sustained during a sports collision, required prompt surgical intervention to restore tendon continuity and joint congruity. Early rehabilitation and an aggressive return-to-sport protocol yielded a successful outcome, with full functional recovery. A literature review reveals limited documentation of similar cases, underscoring the clinical importance of early recognition and operative management in athletes.

## Introduction

Closed FDS injuries are far less common than an FDP avulsion, likely due to its more proximal insertion and protective position relative to the FDP. Isolated rupture of FDS with an intact FDP has been described in prior literature, however the combination of FDS rupture in the setting of a proximal interphalangeal joint fracture dislocation has not. The PIP joint fracture-dislocation suggests a concurrent axial load and dorsal-directed force, which likely occurred simultaneously during the tackle event in this case. Such injuries require prompt intervention to restore joint congruity and prevent long-term complications such as stiffness, arthritis, or swan-neck deformity.^1^ This case report details the presentation, surgical treatment, and outcome of such a combined injury in a young athlete.

## Case presentation

### Patient profile

A 17-year-old right-hand-dominant male presented to the clinic with acute pain, swelling, and deformity of his left middle finger. The injury occurred 3 days prior to presentation during a high school football game when his finger became caught in another player’s jersey. The patient reported a sudden tearing sensation and pain resulting in a clear deformity. He was otherwise healthy, highly active, and had no prior history of trauma to his hand.

### Clinical findings

Inspection demonstrated significant swelling and effusion at the left middle finger PIP joint with notable deformity. There was diffuse tenderness to palpation and a painful active and passive range of motion limited to 0–30 degrees. Joint instability was noted on stress testing. No open wounds were present. Sensory examination was normal with intact sensation on the radial and ulnar aspects of the digit. Capillary refill was brisk. There were no signs of sensory compromise.

### Imaging

**Figure 1:**
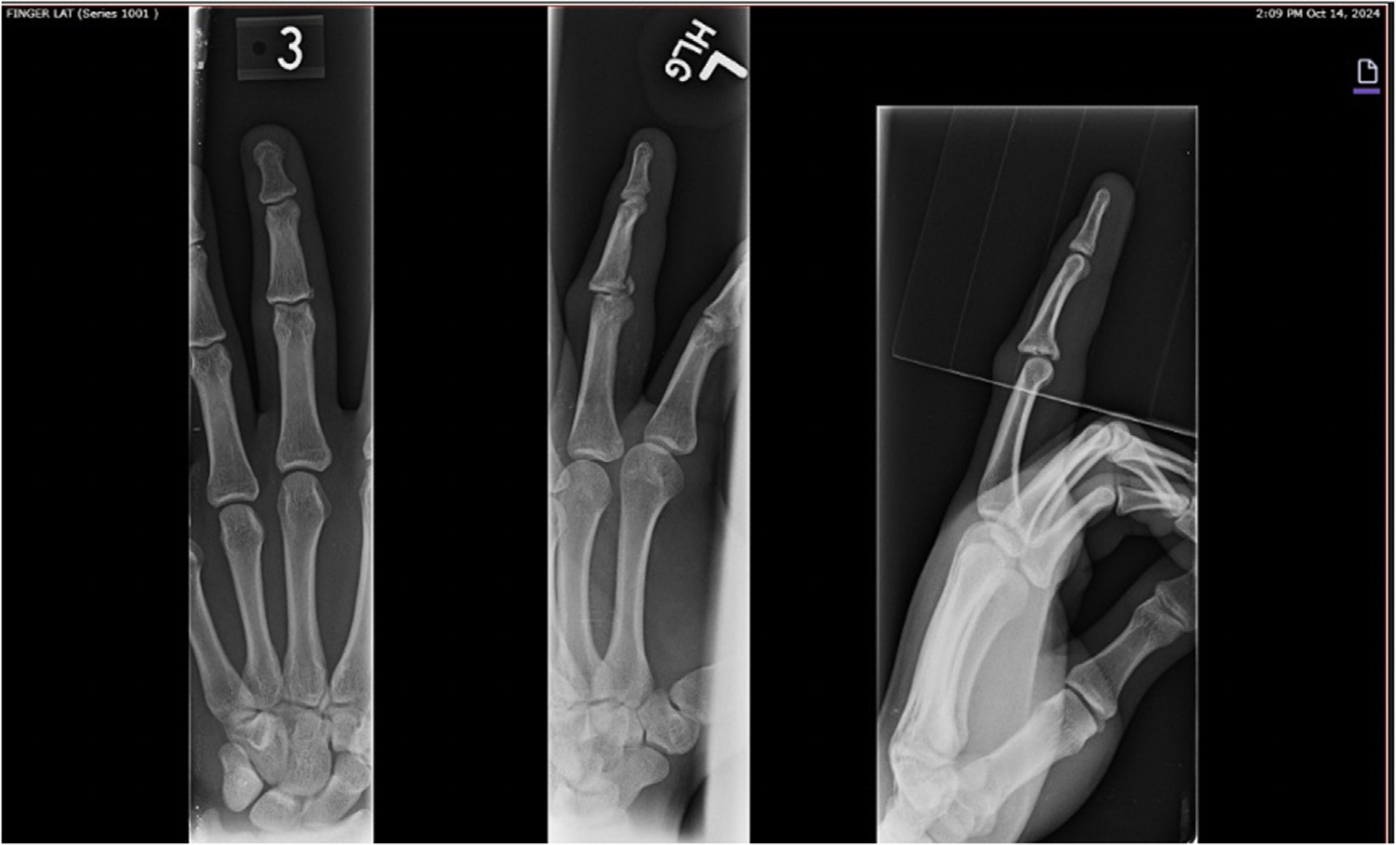
Preoperative imaging.

Three-view radiographs of the left hand showed a comminuted articular fracture of the base of the middle phalanx involving approximately 50 % of the articular surface, along with dorsal subluxation of the PIP joint. Findings were consistent with a dorsal fracture-dislocation as seen in Figure 1.

### Surgical management

The patient underwent urgent surgical repair under sedation and local anesthesia. A standard Brunner incision was made over the PIP joint. On exploration, significant fluid was encountered within the flexor sheath. A partial tear at the proximal aspect of the A4 pulley was identified.

An abnormality was then identified, as the FDS tendon was absent in the field, and only the FDP was present. The insertion site on the distal phalanx revealed bare bone at both the radial and ulnar slip footprints. Subsequently, the FDS tendon was retrieved proximally and tagged for later reattachment. The remainder of the surgical approach progressed as planned, and the PIP joint was exposed in a “shotgun” fashion. Impacted articular fragments were visualized and carefully disimpacted using a small elevator and dental pick. After confirming anatomical reduction, three 0.028-inch K-wires were placed for temporary fixation. These were sequentially overdrilled and replaced with interfragmentary screws: one 1.0 mm screw centrally, and two 1.3 mm screws radially and ulnarly.

**Figure 2:**
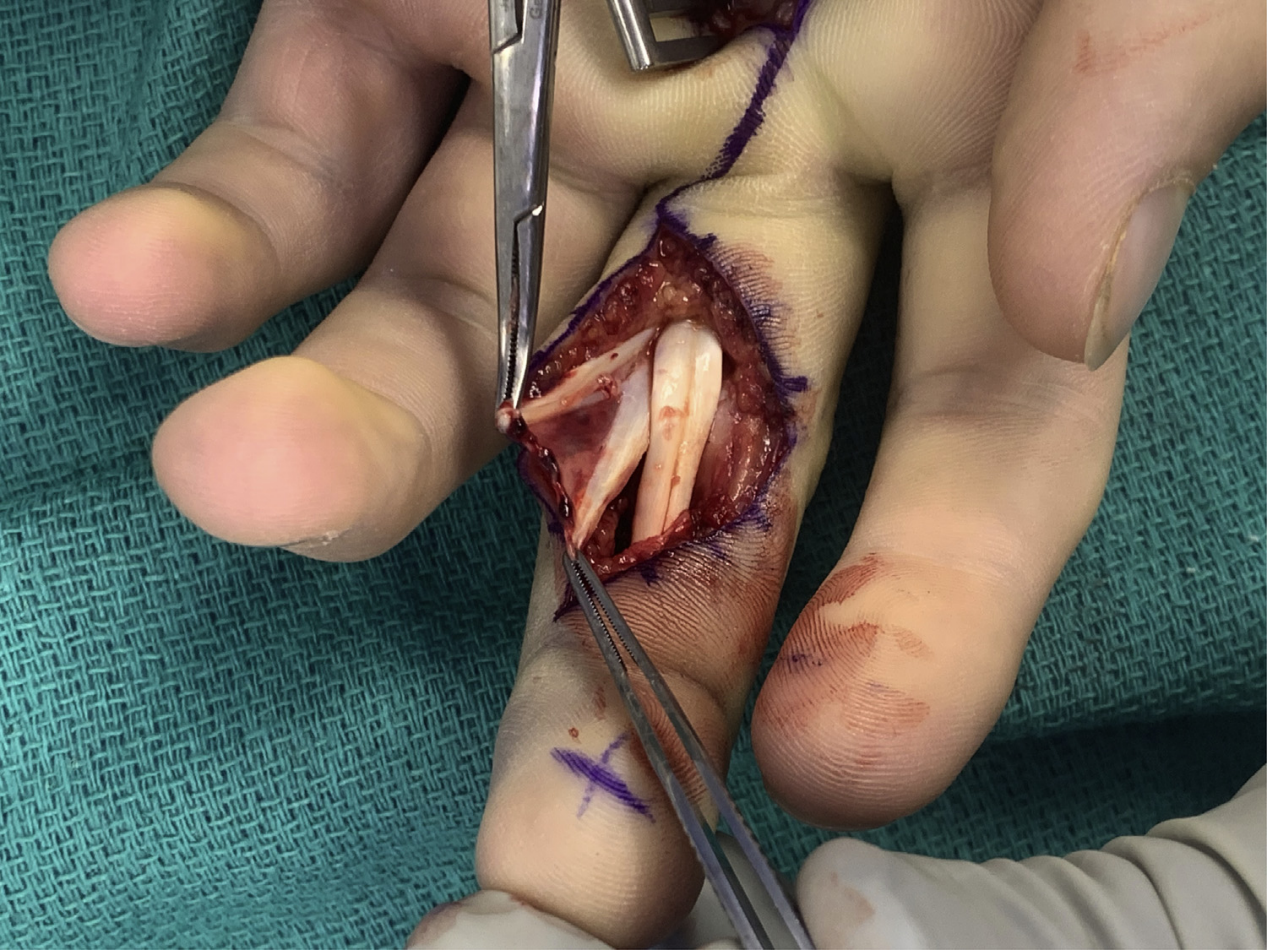
Flexor digitorum superficialis (FDS) tendon avulsion.

After careful reduction of the joint, the flexor tendon avulsion was addressed, visualized in Figure 2. A 1.0 mm anchor was placed at both the ulnar and radial FDS insertion footprints, allowing anatomic repair of the tendon. Intraoperative fluoroscopy confirmed proper joint alignment and stable fixation. Passive ROM demonstrated good tendon gliding with no gapping or instability. Hemostasis was achieved, and the incision was closed with 4–0 nylon sutures. A xeroform dressing and dorsal blocking splint were applied.

### Postoperative course and outcome

#### Two-week follow-up

The patient reported well-controlled pain and was compliant with hand therapy. The incision was clean and well-healed. Sutures were removed. Composite flexion was <1 cm from the fingertip to palm, though terminal extension was deferred due to recent repair. Sensory and motor function remained intact. Radiographs showed stable screw placement and joint congruity.

#### Six-week follow-up

**Figure 3:**
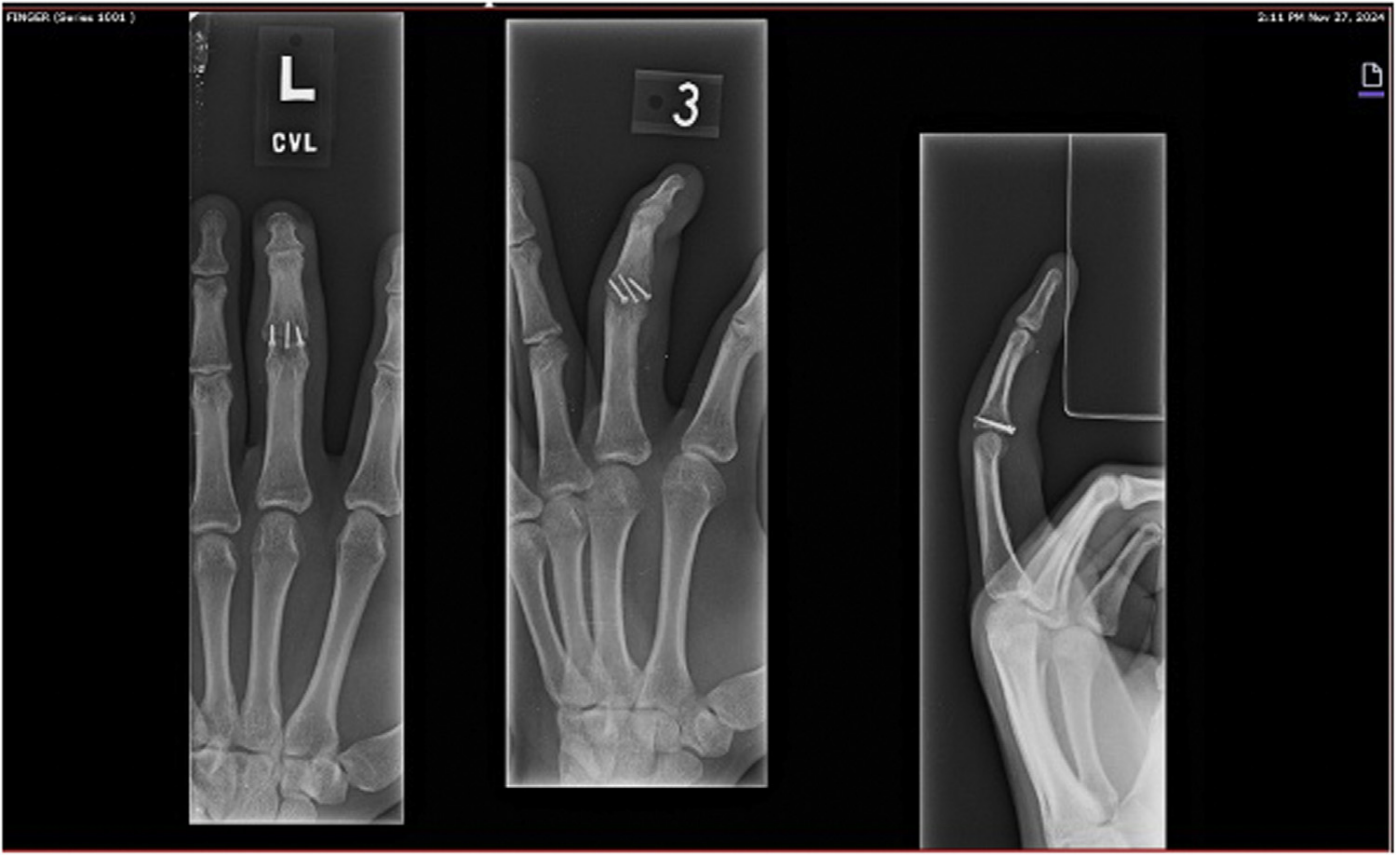
Six-week postoperative imaging.

The patient had resumed basketball and weightlifting without pain. He reported mild swelling interfering with wearing a baseball glove. Examination showed well-healed incisions and full flexion of all digits. A slight PIP flexion contracture persisted. Radiographs confirmed continued healing and stable fixation. Clinical and radiographic union was evident as seen in Figure 3, and the patient was cleared to return to all sports without restriction.

### Therapy course

The patient completed 20 hand therapy sessions including therapeutic exercises, functional activities, orthotic fabrication and fitting, and self-care education as seen in Table 1. A custom dorsal blocking orthosis was used postoperatively and transitioned to functional splinting as healing progressed.

**Table 1:** Physical therapy progression between initial session and final session.

	First session (October 10, 2024)	Last SESSION (March 3, 2025)
L middle finger MP extension	−30	0
L middle finger MP flexion	85	90
L middle finger PIP extension	−30	−10 (0 at end of session)
L middle finger PIP flexion	85	100
L middle finger DIP extension	0	0
L middle finger DIP flexion	60	85

## Discussion

Closed ruptures of the FDS are rare, and when they occur in conjunction with a proximal interphalangeal (PIP) joint fracture-dislocation, the complexity of diagnosis and treatment significantly increases. This unique combination challenges both hand surgeons and rehabilitation teams due to the mechanical, functional, and anatomical disruption of the finger’s flexor apparatus and joint stability.

There has not been a reported case of an intact FDP with a combination of a FDS avulsion and PIP fracture-dislocation. Initial clinical assessment may be misleading, particularly in high-stakes environments like athletic events, where swelling and pain obscure functional deficits. A meticulous physical exam must assess individual tendon function—often difficult in acute settings. Ultrasound or MRI may aid in confirming tendon ruptures, but plain radiographs are essential for identifying associated bony injuries such as volar lip fractures or dorsal subluxations.^5^ In this case, FDS repair was performed not only to restore tendon continuity but also to enhance PIP joint stability. Given the associated dorsal fracture-dislocation, the FDS served as a dynamic checkrein against recurrent dorsal instability and provided additional protection to the osseous fixation.

Postoperative rehabilitation plays a crucial role in optimizing functional outcomes. Early controlled range of motion is essential to prevent joint stiffness but must be carefully balanced with protection of the repair. This is particularly difficult when both tendons and the joint are injured, as in this case. Close collaboration with hand therapy specialists and adherence to a tailored protocol are critical.

Comparative literature suggests that isolated FDS ruptures are often managed conservatively if no associated fracture is present, but combined injuries such as fracture-dislocations necessitate surgical intervention to restore joint stability and tendon function.^2^^,^^3^ Other cases of flexor tendon avulsions, particularly those involving FDP, have demonstrated poorer outcomes if delayed or inadequately repaired.^4^^,^^6^^,^^7^ The uniqueness of this case, with intact FDP and isolated FDS avulsion, supports the need for individualized surgical planning and early operative repair to optimize results.

Furthermore, the surgical technique utilized—a combination of suture anchor repair and rigid fracture fixation—allowed for early mobilization, reducing the risk of stiffness and improving functional recovery. Similar approaches have been advocated in the literature for complex flexor tendon injuries combined with bony pathology.^8^^,^^9^

The patient’s return to sports without limitation is a testament to the effectiveness of the surgical and rehabilitative protocol, emphasizing the importance of multidisciplinary care in athletes with complex hand injuries. This aligns with recent case series advocating for aggressive early repair and structured therapy to maximize return to function in high-demand populations.^10^^,^^11^

Overall this case represents a rare and severe combination injury of FDS avulsion and a PIP joint fracture-dislocation with an intact FDP in a young athlete. Early recognition and timely surgical management are essential to restore hand function and enable return to sports. Awareness of such injury patterns should prompt thorough evaluation of tendon function and joint integrity in any athlete presenting with significant finger trauma. As such injuries are exceedingly rare, each reported case adds valuable insight into best practices for management and expected outcomes.

Ultimately, prompt surgical intervention with meticulous repair of both tendon and bony components, combined with an individualized rehabilitation protocol, can lead to excellent functional outcomes and return to athletic activities. Further studies and case reports are encouraged to better delineate treatment algorithms for these uncommon but challenging injuries.

## Ethical approval

Not required.

## Informed consent

Written informed consent was obtained from the patient for publication of this case report and accompanying images.

## Declaration of competing interest

None declared.

## References

[1] Caggiano N.M., Harper C.M., Rozental T.D. (2018). Management of proximal interphalangeal joint fracture dislocations. Hand Clin.

[2] Bazavar M., Rouhani A., Tabrizi A. (2014). Simultaneous dorsal base fracture and FDP avulsion of distal phalanx of the little finger. Arch Bone Jt Surg.

[3] Boyes J.H., Wilson J.N., Smith J.W. (1960). Flexor-tendon ruptures in the forearm and hand. J Bone Joint Surg Am.

[4] Jordan R.W., Lotfi N., Shyamalan G. (2015). Simultaneous closed rupture of flexor digitorum superficialis and flexor digitorum profundus tendons in the middle finger: a case report. Case Reports Plast Surg Hand Surg.

[5] Johnson M.A., Colville J. (2020). Closed traumatic avulsion of both ring finger flexors with successful primary repair >4 weeks after injury and a review of the literature. J Surg Case Rep.

[6] Ching R.C., Stevenson S. (2020). An unusual pattern of closed flexor tendon avulsion. J Hand Microsurg.

[7] Xarchas K.C., Tilkeridis K., Kitsikidou G., Pelekas S.I., Verettas D.A. (2009). Complete avulsion of the extensor mechanism of a finger with simultaneous dislocation of the proximal interphalangeal joint. Open Orthop J.

[8] Solunke S., Deshmukh A., Nair A., Chopra S., Gupta A., Chaudhary S. (2024). Flexor digitorum profundus avulsion with distal interphalangeal joint dorsal dislocation: a case report. Cureus.

[9] Aharram S., Mounir Y., Benhamou M. (2019). Fracture avulsion of the flexor digitorum profundus tendon: case report. MOJ Clin Med Case Rep.

[10] Soro M.A., Christen T., Durand S. (2016). Unusual closed traumatic avulsion of both flexor tendons in zones 1 and 3 of the little finger. Case Rep Orthop.

[11] Cuggy C., Woods J.F.C., Carr E., Jumper N., Lynch N., Dolan R. (2024). Closed traumatic avulsion of both flexor digitorum tendons: an addition to the Leddy and Packer classification and review of the literature. J Hand Microsurg.

